# Chitosan Films for Microfluidic Studies of Single Bacteria and Perspectives for Antibiotic Susceptibility Testing

**DOI:** 10.1128/mBio.01375-19

**Published:** 2019-08-20

**Authors:** Julie Tréguier, Loic Bugnicourt, Guillaume Gay, Mamoudou Diallo, Salim Timo Islam, Alexandre Toro, Laurent David, Olivier Théodoly, Guillaume Sudre, Tâm Mignot

**Affiliations:** a Laboratoire de Chimie Bactérienne, Institut de Microbiologie de la Méditerranée, CNRS-Aix Marseille University (UMR7283), Marseille, France; b Ingénierie des Matériaux Polymères, Université Claude Bernard Lyon 1, Université de Lyon, CNRS UMR 5223, Villeurbanne, France; c Morphogénie Logiciels SAS, Marseille, France; d Laboratoire de Biologie, Centre Hospitalier de Martigues, Martigues, France; e Laboratoire Adhésion et Inflammation, INSERM U1067, CNRS UMR 7333, Marseille, France; National Institute of Child Health and Human Development (NICHD)

**Keywords:** antibiotic susceptibility testing, chitosan, *Escherichia coli*, *Klebsiella pneumonia*, single cells, microfluidics

## Abstract

Single-cell microfluidics is a powerful method to study bacteria and determine their susceptibility to antibiotic treatment. Glass treatment by adhesive molecules is a potential solution to immobilize bacterial cells and perform microscopy, but traditional cationic polymers such as polylysine deeply affect bacterial physiology. In this work, we chemically characterized a class of chitosan polymers for their biocompatibility when adsorbed to glass. Chitosan chains of known length and composition allowed growth of Escherichia coli cells without any deleterious effects on cell physiology. Combined with a machine learning approach, this method could measure the antibiotic susceptibility of a diversity of clinical strains in less than 1 h and with higher accuracy than current methods. Finally, chitosan polymers also supported growth of Klebsiella pneumoniae, another bacterial pathogen of clinical significance.

## Abstract (precis)

Current microfluidic techniques are powerful to study bacteria and determine their response to antibiotic treatment, but they are currently limited by their complex manipulation. Chitosan films are fully biocompatible and could thus be a viable replacement for existing commercial devices that currently use polylysine. Thus, the low cost of chitosan slides and their simple implementation make them highly versatile for research as well as clinical use.

## INTRODUCTION

In recent years, microfluidics coupled with live-cell imaging has revolutionized bacteriology, testing directly the impact of rapid and controlled environmental transitions on cell physiology. With the advent of superresolution microscopy, the bacterial cell can now be further explored at an unprecedented resolution, tracking cellular processes one molecule at a time (1). The impact of these methods is not limited to basic research because single-cell approaches are unquestionably powerful at determining antimicrobial susceptibility (via antimicrobial susceptibility testing [AST]) in record time (2, 3).

However, a major technical bottleneck with the implementation of single-cell approaches for superresolution or AST is the immobilization of bacterial cells. Agar surfaces have been widely used and support the growth of a wide range of bacterial species. However, this method has several limits:

1. Agar surfaces have important limitations for high-end microscopy (HEM) methods (including all the single-molecule microscopy techniques photoactivated localization microscopy [PALM], stochastic optical reconstruction microscopy [STORM], and stimulated emission depletion [STED]): drift of the agarose pad due to dessication and local changes in surface flatness as well as high background levels (1). In addition, agar surfaces are not compatible with total internal reflection fluorescence microscopy (TIRFM), which requires a flat, fully transparent surface.
2. Because the adhesion of bacterial cells to agar surfaces is generally weak, these surfaces cannot be manipulated in aqueous environments, and the experimental conditions are generally set by diffusion through the agar substrate. However, this approach does not allow rapid changes of the medium or injection of chemicals, and thus the kinetics and precise dose-dependent effects are poorly controlled (3, 4).

Alternative methods have remedied these issues by growing bacteria immediately in contact with a glass surface. Because most bacteria do not directly adhere to glass, immobilization procedures are required, which include direct physical immobilization of the bacteria in microchannels or glass functionalization by adhesive polymers. The use of microchannels is certainly compatible with HEM, and it allows fast AST with high accuracy (2, 5). However, this method requires polydimethylsiloxane-based soft lithography, which although it is becoming quite standard, still requires expert handling to be used for the study of a given bacterial species. For AST in clinical contexts, these technical bottlenecks render these approaches difficult due to the high need for calibration and automation at the hospital.

Alternatively, bacterial adhesion on glass can be obtained by functionalizing a glass slide with adhesive polymers/molecules. This approach can also be difficult because the polymer must be fully biocompatible, and the functionalization procedure and surface chemistry can be complex. A number of coatings employing extracellular matrix proteins are available for eukaryotic cells, but the choice for polymers biocompatible with bacteria is limited. Cationic polymers such as polylysine bind glass surfaces effectively and promote adhesion of a wide range of bacterial species. However, polylysine also generates cell envelope stress and has been shown to dissipate/diminish the membrane potential in several Gram-negative or Gram-positive species (e.g., Escherichia coli or Bacillus subtilis [6–8]). For clinical microbiology applications, this issue is particularly sensitive because changes in the membrane potential can directly affect antimicrobial susceptibility (9) and thus produce false-negative or, even worse, false-positive results in AST. Polylysine coating is nevertheless exploited by the so-called “Accelerate Pheno system” (APS [Accelerate Diagnostics]), the first commercial AST system with single-cell resolution, which is currently being tested in hospitals (10, 11). Specifically, and in the absence of a more-adapted coating, the APS uses polylysine and indium tin oxide (ITO) facilitated gel electro-filtration to immobilize bacteria (12). This procedure, however, must affect the accurate measurement of MICs because decreasing the proton motive force can artificially result in increased antibiotic resistance for some classes of antibiotics (9). Adapting new surface coatings for devices such as the APS thus holds promise for clinical use.

This work originated from the observation that chitosan polymers can support bacterial adhesion and motility on surfaces (in the case of Myxococcus xanthus and Bacillus subtilis [1]). However, these procedures were derived from commercial chitosan batches, which very poorly supported bacterial growth and showed low reproducibility due to batch-to batch variations. This is in fact not surprising because commercial chitosan is essentially produced from chitin hydrolysis in weak acids, and the resulting chitosan chains are not characterized precisely in terms of average molar mass (*M*_w_) and degree of acetylation (DA [described below]). Knowing these parameters and their biological impact is in fact crucial because chitosan polymers have a wide range of biological properties, depending on their composition; critically, bacteriostatic effects have been described for chitosan classes (13). Here, we investigated if biocompatible chitosan polymers of measured chemical composition could support bacterial growth when inserted into commercial microfluidic chambers. We thus identified a specific chitosan polymer (with a high DA) and a new controlled functionalization procedure that can support the growth of E. coli during multiple generations without any effects on bacterial fitness. Using clinical E. coli strains obtained from intestinal and/or urinary tract infections (ITIs/UTIs) of known antibiotic susceptibilities, we showed that chitosan-coated slides (CCSs) allowed fast, direct determination of AST. Finally, CCSs can be derived to promote growth of other so-called ESKAPE pathogens (Enterococcus faecium, Staphylococcus aureus, Klebsiella pneumoniae, Acinetobacter baumannii, Pseudomonas aeruginosa, and *Enterobacter* species), such as K. pneumoniae, which also raise significant problems for antibiotic treatment (14). We conclude that chitosan-based functionalization procedures are promising for their application in bacterial single-cell studies for basic research and also potentially in clinical contexts.

## RESULTS

### Functionalization of glass slides with chitosan polymers.

**FIG 1:**
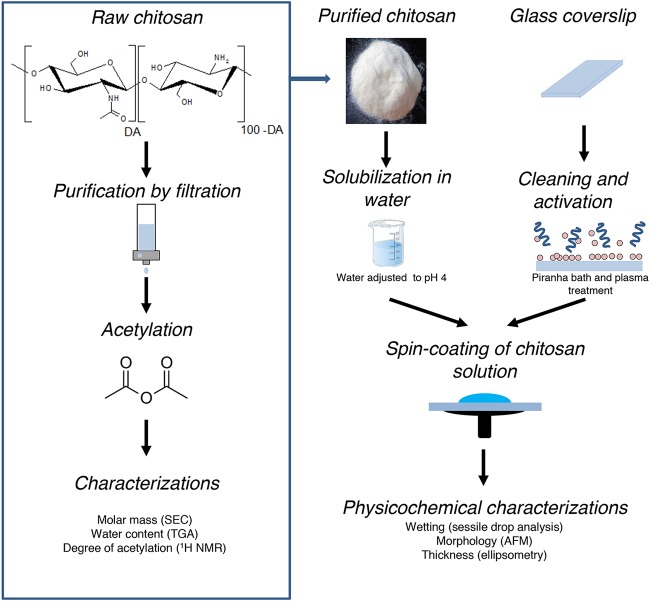
Functionalization of glass slides with chitosan polymers. Shown are procedures for chitosan preparation, glass surface modification, and characterization of chitosan layers. See Materials and Methods for details.

Chitosan is a linear polysaccharide composed of randomly distributed β-(1→4)-linked d-glucosamine and *N*-acetyl-d-glucosamine units (Fig. 1). Its physicochemical properties are highly dependent on its macromolecular parameters (i.e., *M*_w_ and DA). Control of these parameters is needed to ensure robustness when studying the physicochemical and biological behavior of chitosan polymers. Indeed, growth and motility were not always reproducible when glass slides were coated with raw commercial chitosan from commercial stocks, of which precise characterization is difficult because it contains chains of variable DA, molar masses, and statistical distributions of the acetyl groups.

Consistent with this, size exclusion chromatography (SEC) analysis (SEC coupled with multiangle laser light scattering with refractive index detection [SEC-MALLS/RI]) performed on chitosan from a commercial source (Sigma-Aldrich [see Materials and Methods]) revealed an important dispersity (*Ð*) in polymer chain length (*Ð* = 2.65). It is essential to control the dispersity of chitosan chains because slight variations in molar mass and DA of chitosan polymers can be associated with a wide range of biological responses: eukaryotic cell adhesion, wound healing, and even bacterial stasis and lysis (15, 16). In general, it is mandatory to determine and control each molecular parameter in order to understand their impact on bacterial physiology and to ensure reproducibility of our experiments.

To this aim, we first generated a large library of chitosan polymers with various DA and molar masses (17) (Chito-library [see Materials and Methods]). Different molar masses (low *M*_w_ of 156 kDa and high *M*_w_ of 557 kDa) were obtained by selecting chitosan from different sources (shrimp and squid, respectively). To control the degree of acetylation, the polymer chains were reacetylated *in vitro* to produce DA ranging from 1% to 52.6% (18). Each polymer was characterized by size exclusion chromatography (SEC) to control its molar mass, by ^1^H nuclear magnetic resonance (NMR) to measure its DA, and by thermogravimetric analysis (TGA) to evaluate its water content (Fig. 1) (see Materials and Methods).

Flat and homogeneous layers of polymer in the nanometer range (i.e., thickness of >15 nm and <150 nm) were obtained by spin-coating of chitosan solutions with controlled concentration and pH (Fig. 1) directly on boro-silicate glass coverslips for bacterial single-cell studies (Fig. 1). The thickness, uniformity, wettability, and morphology of chitosan ultrathin films prepared from the Chito-library were systematically examined by ellipsometry, tensiometry, optical microscopy, profilometry, and atomic force microscopy (AFM) (Fig. 1). The detailed information on the physicochemical properties of the chitosan thin films will be described elsewhere in a specialized dedicated publication. Whatever the formulations studied, the thickness and wettability of chitosan layers were highly reproducible (e.g., 23.3 ± 1.3 nm and 37.8 ± 1.2°, respectively, for chitosan formulation of DA = 52.2%, [*c*] = 0.67%, and *M*_w_ = 156 kDa, with *n* = 10), with a root mean square (RMS) roughness of <1 nm.

We thus successfully generated homogeneous CCSs of known polymer molar mass, DA, and thickness. By varying the chitosan macromolecular parameters and chitosan solution characteristics, more than 50 different chitosan coatings were thus prepared to be screened for their ability to support bacterial proliferation.

### Specific chitosan polymers promote adhesion and normal growth of E. coli cells.

We next tested the ability of the various types of CCSs to support the adhesion and ultimately growth of the main laboratory E. coli K-12 strain. To perform this screening, we divided our CCS library into nine representative subclasses, based on source, DA, and additional treatments (Table 1). Each CCS type was then mounted at the bottom of a microfluidic cassette and tested for E. coli adhesion and growth (see Materials and Methods).

**TABLE 1:**
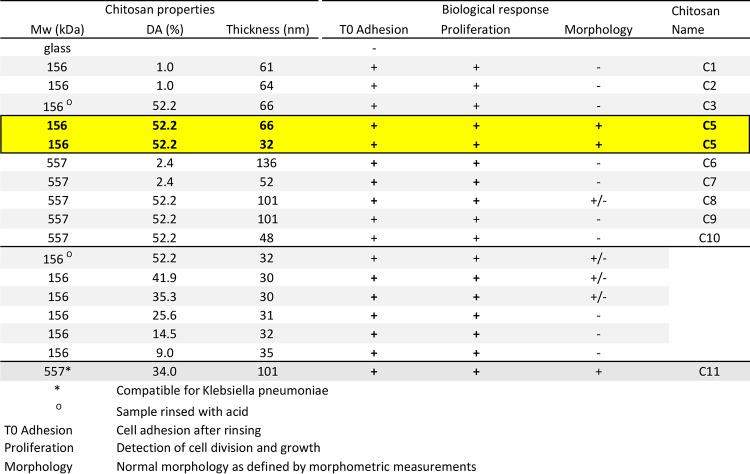
Chitosan types and adhesion and proliferation of E. coli and K. pneumonia

**FIG 2:**
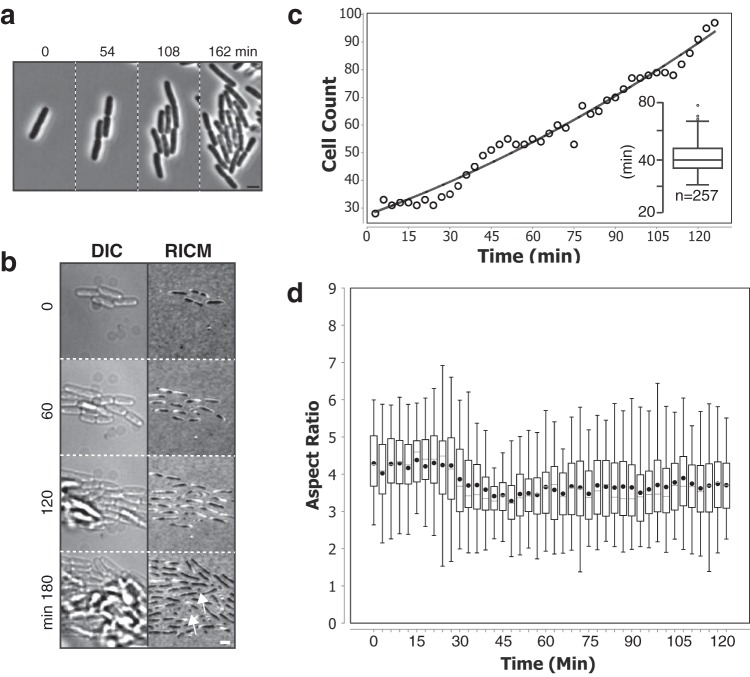
Growth of E. coli on selected C5 chitosan slides. (a) Growth of E. coli K-12 on C5. Shown are snapshots separated by 54 min after growth initiation (left panel). See the associated Movie S1 in the supplemental material for the full time lapse. Scale bar = 2 μm. (b) Adhesion of E. coli on C5 as measured by reflection interference contrast microscopy (RICM). E. coli is shown by Nomarski imaging (differential inference contrast [DIC], left panels) revealing the three-dimensional organization of the E. coli microcolony and by RICM to reveal the adhesion sites (observed as dark areas, right panels). Note that the cells remain tightly adhered to the chitosan surface even at the latest time points when the microcolony clearly expands above the focal plane. White arrows point to areas where the cells remain adhered by the cell pole only, allowing them to grow away from the chitosan surface. See associated Movie S2 for the full time lapse. Scale bar = 2 μm. (c) Growth of E. coli K-12 on C5. Shown is an exponential fit of the number of cells as a function of time. (Inset) Growth rate distribution on C5. (d) Morphology of E. coli on C5 over time. The aspect ratios were determined from phase-contrast images of adhered cells and correspond to the ratio between the lengths of the long axis and the short axis of the cell.

We found that while Luria-Bertani (LB)-grown E. coli cells did not adhere to uncoated glass slides, they adhered to all CCS types, showing that chitosan can indeed promote adhesion of E. coli. However, while E. coli cells did generally proliferate on these surfaces, growth was frequently abnormal, as evidenced by cell filamentation and morphological aberrations (see Fig. S1A in the supplemental material). Nevertheless, two types of CCSs obtained with one chitosan, C5 (DA = 52.2%, *M*_w_ = 156 kDa), and two thicknesses of 32 and 66 nm supported normal growth (Fig. 2a and Table 1). To further characterize this chitosan class, we tested 156-kDa chitosans of various DA and found that DA of ≥50% were required for normal growth (Table 1). In addition, formulation was important because acid rinsing negatively impacted the biocompatibility of the procedure (Table 1).

10.1128/mBio.01375-19.1 FIG S1 (A) Morphological aberrations on chitosan. Shown is E. coli K-12 growth on C8 (Table 1). Note that the cells show abnormal twisted filaments that gradually detach over time. Scale bar = 2 μm. (B) RICM of E. coli on torsion-inducing chitosan. On C8, RICM reveals that E. coli K-12 cells are mostly attached by the cell poles, explaining their twisted shape due to the combined growth and local adhesion points. Scale bar = 4 μm. (C) C5 shows no long-term toxicity. Shown are E. coli K-12 cells that resume growth after being left for 3 days in exhausted medium and with addition of fresh medium. Cell growth resumes normally. Scale bar = 8 μm. Download FIG S1, PDF file, 1.4 MB. Copyright © 2019 Tréguier et al. 2019 Tréguier et al. This content is distributed under the terms of the Creative Commons Attribution 4.0 International license.

10.1128/mBio.01375-19.5 MOVIE S1 Growth of E. coli K-12 on C5. Download Movie S1, AVI file, 0.1 MB. Copyright © 2019 Tréguier et al. 2019 Tréguier et al. This content is distributed under the terms of the Creative Commons Attribution 4.0 International license.

10.1128/mBio.01375-19.6 MOVIE S2 RICM of E. coli K-12 on C5. Download Movie S2, AVI file, 2.3 MB. Copyright © 2019 Tréguier et al. 2019 Tréguier et al. This content is distributed under the terms of the Creative Commons Attribution 4.0 International license.

We next characterized the ability of C5 to promote adhesion and growth in detail. E. coli K-12 cells formed monolayer microcolonies and could be monitored for up to 6 generations, after which the cells started growing above the focal plane defined by the glass slide. Expansion of the E. coli microcolony in three dimensions (3D) could occur because tight adhesion of the monolayer forced the daughter cells to grow away from the immediate surface, which has been shown to act as driving force for bacterial colony and biofilm development (19). To test this possibility, we analyzed E. coli cell adhesion to C5 by reflection interference contrast microscopy (RICM), a technique that allows imaging of intimate cell contacts with glass surfaces (20). RICM revealed that each cell remained in close contact with the glass surface by adhering along its axis. Surface escape was due to steric constraints and vertical growth of bacteria adhered via their cell poles (Fig. 2b; see Movie S2 in the supplemental material). In contrast, when we performed RICM on a CCS type that created abnormal cell torsions, it was apparent that the dividing cells only adhered via the cell poles, explaining cell detachment and the emergence of torsions (Fig. S1B). Consistent with the RICM results, E. coli cells remained attached to C5 even when subjected to shear stress of up to 12 dyn/cm^2^ (which is comparable to shear stress generated in the aorta [21]; see Materials and Methods).

We next tested whether C5 created detectable stress on E. coli K-12 growth. E. coli K-12 cells grew exponentially, with an average 39-min generation time, similar to the generation of E. coli grown under agitation in liquid culture at room temperature (Fig. 2c). Cell morphology, measured by the aspect ratio (length/width), remained stable over time, showing that it was not affected on C5 (Fig. 2d). Finally, to test whether C5 generates long-term cellular defects, we allowed E. coli cells to develop on C5 until they reached stationary phase and became quiescent for 3 days. These cells resumed growth normally after fresh medium was injected, showing that long-term exposure to C5 does not affect cell viability (Fig. S1C). Although all experiments were performed at room temperature for practical reasons (to avoid the use of a thermo-controller system), C5 also supported growth of E. coli at 37°C without detectable fitness cost compared to liquid-grown cultures (generation time = 22 ± 2 min; *n* = 308). We conclude that C5 is a well-adapted chitosan to grow E. coli K-12 cells on a glass surface in microfluidic chambers.

### CCSs allow fast antibiotic susceptibility testing.

**FIG 3:**
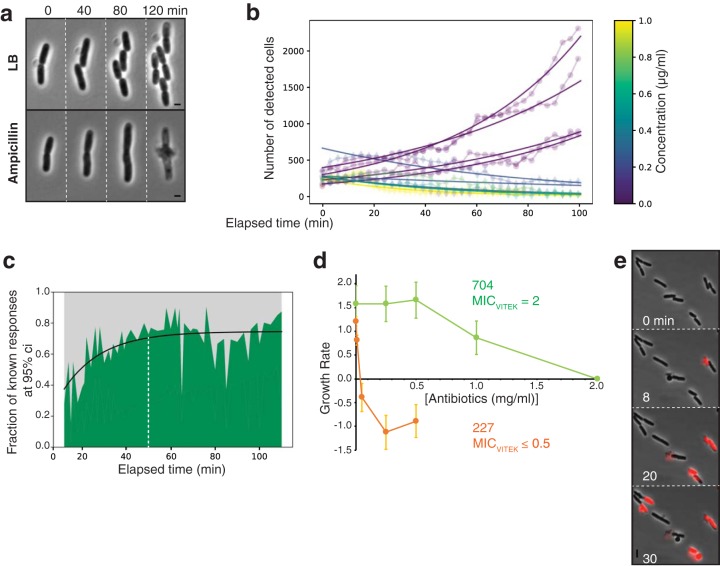
C5 CCSs can be used for fast AST of E. coli clinical strains. (a) Ampicillin treatment is effective on C5. Note the characteristic ampicillin-induced morphological transitions, cell elongation, and formation of a septal lytic “bubble” that precludes cell death. See associated Movie S3 for the full time lapse. (b) Trained detection of ertapenem (ETP) effects on growth of E. coli clinical strains. Shown are measured growth curves for strain UTI227 with various concentrations of ETP. Fitted growth curves computed from the number of detected cells across time are color coded with respect to the ETP concentration. For each curve, the plot symbol is circular if the cells survive and diamond shaped if the cell population stalls or shrinks due to cell death. (c) Estimation of the minimal diagnostic time. We performed an estimation of the growth rates for various time spans for all assays and determined for each time span the fraction of assays for which the response could be ascertained with a 95% confidence interval (Ci). (d) Comparison of the MICs as measured on CCSs with MICs obtained at the hospital. The MIC is determined for growth rates of ≤0 obtained at given antibiotic concentrations. Note that the hospital (Vitek) and CCS-determined MICs for amdinocillin are similar for UTI704, but that the CCS method measures MICs as low as 0.05 for UTI227 in the presence of ertapenem. (e) Detection of cell death by propidium iodide (PI) staining. PI only stains the bacterial DNA of permeable dead cells, which provides fast and sensitive quantification of MBCs. See associated Movie S4 for a typical time lapse.

Beyond their obvious use in research applications, CCSs could provide a fast and reliable tool for AST. For this, CCSs should be significantly faster than and at least as reliable as currently used methods. To test this, we incubated E. coli K-12 cells on C5 and injected ampicillin, which rapidly resulted in the typical cell elongation and formation of a bulge in the septal zone that precludes cell lysis (Fig. 3a; see Movie S3 in the supplemental material). The approximate time-to-death (*T_d_*) was ∼120 min, consistent with the kinetics described in other single-cell experiments (22). On C5, ampicillin generates the same cellular defects as in other studies and could thus be used for AST.

10.1128/mBio.01375-19.7 MOVIE S3 E. coli K-12 in the presence of ampicillin. Download Movie S3, AVI file, 0.6 MB. Copyright © 2019 Tréguier et al. 2019 Tréguier et al. This content is distributed under the terms of the Creative Commons Attribution 4.0 International license.

10.1128/mBio.01375-19.8 MOVIE S4 E. coli UTI227 in the presence of ertapenem and PI. Download Movie S4, AVI file, 6.4 MB. Copyright © 2019 Tréguier et al. 2019 Tréguier et al. This content is distributed under the terms of the Creative Commons Attribution 4.0 International license.

We next tested whether antibiotic susceptibility may be determined in less time than the measured *T_d_* (as detected by irreversible cell lysis). Indeed, although E. coli cells lyse after 2 h, the action of ampicillin is first characterized by abnormal cell elongation (Fig. 3a). Thus, early detection of abnormal cell morphologies would provide a fast method to assess the action of ampicillin. To do this reliably and computationally, we designed a machine learning-based morphometric method that discriminates abnormal cell morphologies from WT cell morphologies and detects the effect of antibiotics at different treatment times (see Fig. S2A to C in the supplemental material and see Materials and Methods). Briefly, following segmentation and determination of cell contours, this method allows the direct counting of cells with normal morphologies and thus the determination of growth curves. This approach could readily determine growth curves of an E. coli strain isolated from a urinary tract infection (UTI227) and treated with increasing doses of ertapenem, a relatively broad-spectrum carbapenem standardly used at the hospital (Fig. 3b). Lethal ertapenem effects could be detected with 95% confidence as early as 50 min after addition of the antibiotic (Fig. 3b and c). Thus, combined with our computational detection method, CCS (and here specifically C5) is a promising tool for fast AST.

10.1128/mBio.01375-19.2 FIG S2 (a) Cell contour detection of a dividing bacterium after imaging by 100× phase-contrast microscopy. (B) Quantification of the extracted contour. The orange outline reveals the convex hull of the detected contour. The red and gray dots are convexity defects used to detect the septum. Both red dots are automatically assigned to the septum. Further quantifications were based on the ratio of the area corresponding to the bacterial contour to the area of the minimal rectangle encompassing this contour (rectangular fill). (C) Detection training of abnormal (and thus antibiotic-sensitive) cells. Shown is an outlier detection obtained with the scikit-learn one-class scalable vector machine from the annotated data set. For clarity, we only display the data corresponding to two dimensions: circularity and rectangular fill (see Materials and Methods for other parameters). Blue symbols are classified as normal, and red symbols are classified as abnormal. Overall, there is a 10% rate of false-negatives (normal contours labeled as abnormal) in both the training and test sets. This performance induces that a noise is the growth curves and globally results in a 10% uncertainty in the computed growth rate. This uncertainty (which could be reduced with enhanced segmentation procedures) does not significantly affect the accuracy of MIC measurements. Download FIG S2, PDF file, 0.9 MB. Copyright © 2019 Tréguier et al. 2019 Tréguier et al. This content is distributed under the terms of the Creative Commons Attribution 4.0 International license.

### CCSs can be used to measure the MIC of clinical E. coli isolates.

To test the potential clinical application of CCSs more broadly, we next obtained a collection of 15 clinical isolates derived from UTIs (14 isolates) and an intestinal tract infection (ITI [1 isolate]) and tested their ability to grow on C5. We determined that 70% of the clinical strains adhered and grew normally on C5, but this number could be improved to 85%, if the thickness of C5 were increased to 66 nm (Table 1), showing that thickness is another important parameter to increase the application spectrum of C5 to most E. coli clinical strains.

In current clinical practice, the antibiotic susceptibility of a given bacterial strain is determined by its so-called minimal inhibitory concentration (MIC), which corresponds to the lowest antibiotic concentration that prevents growth. To test if MICs determined on C5 can be directly compared to MICs determined by standard methods, we further selected two clinical strains of known MICs (as determined by Vitek2 [bioMérieux]) for amdinocillin (UTI704 MIC of 2 mg/ml) and ertapenem (UTI227 MIC of ≤0.5 mg/ml) and measured their MICs on C5, extracting growth rates with our computational methods (Fig. 3d). In both cases, the results showed remarkable consistency with the Vitek method, and in fact, the CCS method was more sensitive, allowing us to determine that the UTI227 ertapenem MIC is between 0.01 and 0.05 mg/ml (Fig. 3d and Table 2). To further test the validity of the method, we tested the consistency of the measurements over various ranges of ertapenem concentrations for UTI227 and showed that its amdinocillin MIC on C5 also matches the Vitek-determined MIC (Table 2). Further MIC measurements on additional clinical strains UTI687 and UTI698 of ofloxacin and amdinocillin, respectively, also showed good consistency with Vitek measures (Table 2). In conclusion, CCS appears a promising tool to measure MICs rapidly and accurately in hospitals.

**TABLE 2:**
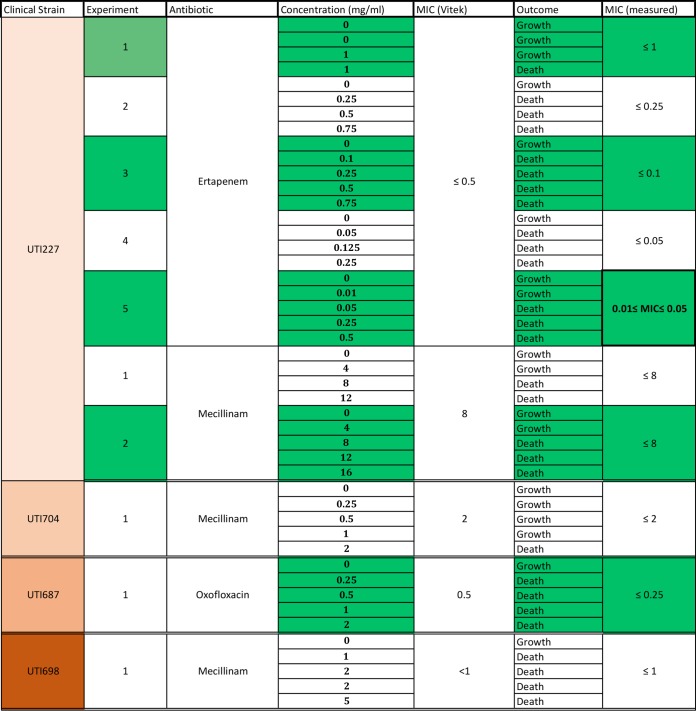
MIC determination in clinical strains

### CCSs can be used to measure the minimal bactericidal concentration of antibiotics.

The MIC does not measure microbial death *per se*, and thus it cannot distinguish bactericidal from bacteriostatic effects. This can be problematic for treatment, especially since it was discovered that antibiotic treatment can induce bacterial persistence, a seemingly dormant state that could be associated with chronic infections (23). Using the minimal bactericidal concentration (MBC), the lowest antibiotic concentration resulting in bacterial death, would in general be more appropriate, but this is a highly time-consuming procedure because it requires regrowth of the bacteria after antibiotic treatment. However, as we show above, the CCS technology allows direct observation of E. coli cell lysis in the presence of ampicillin and is therefore a potential tool to determine the MBC of an antibiotic directly and rapidly (Fig. 3a). In addition, the microfluidic environment of CCS allows detection of cell death: for example, using dyes such as propidium iodide (PI) that only bind the bacterial DNA if the bacterial membrane is irreversibly altered. The use of PI can improve detection sensitivity, especially if the detection method is automated. Indeed, addition of PI to E. coli cells treated with ertapenem allowed detection of cell death on C5, suggesting that this method could be used to determine MBCs in clinical contexts (Fig. 3e; see Movie S4 in the supplemental material).

### CCSs can be adapted to promote surface growth of K. pneumoniae.

**FIG 4:**
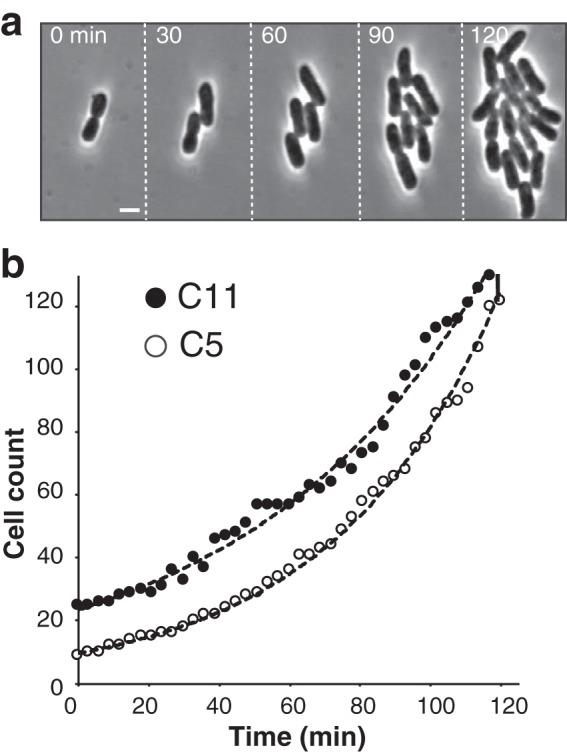
Klebsiella pneumoniae grows on CCSs. (a) Growth of Klebsiella pneumoniae on C5. Shown are snapshots separated by 30 min after growth initiation (left panel). See associated Movie S5 for the full time lapse. (b) Growth of Klebsiella pneumoniae on C5 and C11. The number of cells as a function of time and the corresponding exponential fits are shown.

Next, we were interested in testing whether C5 could be useful to study other clinically relevant pathogens. In particular, and along with E. coli strains, K. pneumoniae is a member of the so-called ESKAPE pathogens, characterized by the high resistance of clinical strains to antimicrobial compounds and thus a growing concern in hospital environments (14). Indeed, K. pneumoniae could readily grow on C5 (but at a 66-nm thickness), with normal morphology and generation time (∼40 min [Fig. 4a and b; see Movie S5 in the supplemental material]). Thus, C5 is a versatile substratum for bacterial adhesion and could be used in hospitals for AST of ESKAPE pathogens.

10.1128/mBio.01375-19.9 MOVIE S5 K. pneumoniae growth on C5. Download Movie S5, AVI file, 8.5 MB. Copyright © 2019 Tréguier et al. 2019 Tréguier et al. This content is distributed under the terms of the Creative Commons Attribution 4.0 International license.

We next wondered if additional “*Klebsiella-*compatible” chitosans could be identified. As discussed above, most tested chitosan polymers are not compatible with E. coli K-12, and it could be interesting to identify “species-specific” polymers for AST. To do this, we further screened the Chito-library and successfully identified one additional CCS type, C11 (DA = 34.0%, *M*_w_ = 557 kDa, and thickness of 101 nm [Table 1]), that also supported *Klebsiella* adhesion and growth without detectable effect on bacterial fitness (Fig. 4b; see Fig. S3 in the supplemental material). Importantly, C11 did not support growth of E. coli. In total, the results suggest that CCS is adaptable to the study of multiple bacterial species and that depending on their chemical structure, chitosan substrates can be derived either to support adhesion and growth of multiple bacterial species or more specifically to grow a given bacterial species or even perhaps a strain.

10.1128/mBio.01375-19.3 FIG S3 Growth of Klebsiella pneumoniae on C11. Shown are snapshots separated by 30 min after growth initiation (left panel). Scale bar = 2 μm. Download FIG S3, PDF file, 0.6 MB. Copyright © 2019 Tréguier et al. 2019 Tréguier et al. This content is distributed under the terms of the Creative Commons Attribution 4.0 International license.

## DISCUSSION

In this work, we report a new glass functionalization procedure that supports bacterial adhesion and growth without any detectable physiological stress, contrarily to currently used polycationic polymers such as polylysine. This technique allows studies of bacteria at the single-cell level in simple microfluidic devices without the need of complex lithography or alternative physical immobilization techniques. Because the chemistry of the chitosan polymers and glass functionalization procedures are well established, the method is robust and highly reproducible. Moreover, CCSs are long-lived, and their integrity is not altered after storage of up to 6 months. Thus, CCSs are highly versatile and provide a viable alternative to other and often more technically challenging microfluidic single-cell approaches.

Although we characterized one CCS type in detail and showed its potential for studies of E. coli and K. pneumoniae for basic and clinical purposes, we also show here that CCSs can be derived for the studies of multiple strains and species—in particular, ESKAPE pathogens. We also performed preliminary tests of the ability of C5 to promote growth of a wide range of Gram-negative and Gram-positive bacteria. In our hands, C5 supported growth of Vibrio cholerae, Myxococcus xanthus, Mycobacterium smegmatis, and Pseudomonas aeruginosa (and also supported *Pseudomonas* twitching motility), but cell adhesion for these species was arguably not optimal. Nevertheless, CCSs could be optimized for these species by testing different C5 thicknesses or, alternatively, by isolating other CCS types as we performed for Klebsiella pneumoniae.

The variable effects of chitosan between species and even within species is not too surprising because the biological properties of chitosan can vary widely based on composition and formulation. For example, chitosan polymers of large size (>550 kDa) and high DA (>50%) are known to exert bacteriostatic effects on some bacteria (15). In addition, adhesion likely depends on the surface properties of the bacteria. In E. coli, phenotypic and genotypic diversity is very wide (24), and thus it is possible that some isolates fail to adhere (albeit a minority) because they have different surface properties (for example, if they carry particular lipopolysaccharide [LPS] O antigens). An interesting avenue for future developments will be to test whether composite CCSs made from several chitosan polymers increase the array of species and strains that may be grown on a single type of slide.

The applications of CCSs in the field of bacterial cell biology are evident as such technology supports studies of any cellular processes, including cell division, but also perhaps studies of more complex population structures such as microcolonies, biofilms, and communities. Using a collection of E. coli strains, we typically observe that the bacteria first proliferate in two dimensions, which we have shown by RICM occurs due to tight adhesion. The bacteria eventually proliferate away from the surface when space becomes a limiting factor for proliferation (Fig. 2b). However, we also observed that some E. coli strains colonize the entire surface in 2D and thus form a single-layer biofilm (see Fig. S4 and Movie S6 in the supplemental material). The formation of E. coli microcolonies on a surface has been shown to depend on both adhesion strength and preferential adhesion of the polar regions (which we also observe here in Fig. 2b) (25). Thus, it is likely that expanded microcolonies are obtained, depending on adhesion strength. Screening conditions that support biofilm formation for a particular strain could be achieved by defining a compatible adhesion range, by modulating the ionic strength of the medium, and/or by changing the chitosan thickness, molar mass, and DA.

10.1128/mBio.01375-19.4 FIG S4 Single-layer colonization of C5 by E. coli clinical strains. Shown is the monolayer development of UTI698. Time points are separated by 54 min. Scale bar = 2 μm. See also associated Movie S6. Download FIG S4, PDF file, 0.8 MB. Copyright © 2019 Tréguier et al. 2019 Tréguier et al. This content is distributed under the terms of the Creative Commons Attribution 4.0 International license.

10.1128/mBio.01375-19.10 MOVIE S6 E. coli monolayers on C5. Download Movie S6, AVI file, 5.9 MB. Copyright © 2019 Tréguier et al. 2019 Tréguier et al. This content is distributed under the terms of the Creative Commons Attribution 4.0 International license.

The search for rapid phenotypic assays to determine antibiotic susceptibility is now a global priority to save on the use of broad-spectrum antibiotics and limit the spread of multiple-antibiotic resistance in hospitals (26). In current clinical practice, AST is generally performed using semiautomated methods that measure growth in bulk cultures in liquid (i.e., Vitek [27]) or solid media. These methods only yield MIC estimates, and the more accurate methods (i.e., antibiotic gradients or E-tests [28]) are time-consuming and costly. Moreover, all of these phenotypic antibiotic susceptibility tests require from 18 to 24 h to provide an estimate of antibiotic susceptibility. Single-cell microscopy approaches are powerful alternatives because they measure MICs as well as MCBs directly, more precisely, and sometimes in less than 30 min—for example, in microchannel chips (2, 29). This technology, however, suffers from important drawbacks linked to sophisticated manipulation and high species-specific use, making its generalization in clinical practice difficult. Also, this method precludes morphometry analysis because the bacteria are maintained in channels that directly constrain their shape. Direct morphometry analysis for rapid AST has shown promising results on bacteria embedded in agarose (3). However, in this case, the antibiotics were added indirectly by diffusion through the agarose, making it difficult to control the exact concentrations and potentially slowing their action. In this context, the CCS method could provide an interesting alternative as we have shown that it can be applied reliably for two major ESKAPE pathogens and it combines the advantages of both approaches described above, allowing direct antibiotic injection and morphometric analyses. The CCS method is more sensitive and ∼10 to 20 times faster than traditional plate assays (here 50 min). A machine learning-based computational approach appears promising to measure MICs in an automated fashion.

For wide use, the CCS method comes at low cost, but it requires technical expertise to prepare specific chitosan polymers and functionalize glass slides with films of calibrated thickness. The process is currently being examined for commercial distribution (30), which could overcome this bottleneck and allow wide dissemination. For clinical use, the method will need to be tested for its compatibility with other pathogens and at much higher throughput. Nevertheless, the results reported here establish a proof of principle that CCS application for MIC determination is feasible.

## MATERIALS AND METHODS

### Materials.

Chitosans with low degrees of acetylation (DA) and different molar masses (*M*_w_) were purchased from Mahtani Co., Ltd.: they included a medium-molar-mass chitosan (chitosan_156_: DA, 1.0%; *M*_w_ = 156.1 kDa/mol; *Đ *=* *1.78; batch 243) and a large-molar-mass chitosan (chitosan_557_: DA, 2.4%; *M*_w_ = 557.2 kDa/mol; *Đ *=* *1.39; batch 114). They were reacetylated to DA ranging from 1 to 52.6% using a procedure previously described (22). Acetic acid (AcOH [99%]), hydrogen peroxide (40% [wt/wt]), sulfuric acid (96% [wt/wt]), hydrochloric acid (HCl [37%]), 1,2-propanediol (99%), and ammonium hydroxide (28%) were purchased from Sigma-Aldrich. Sterile and nonpyrogenic water was purchased from Otec. Silicon wafers (doped-P bore, orientation {100}) were purchased from Siltronix, and glass coverslips (75 by 25 by 0.17 mm^3^; no. 1.5H D263 Schott glass) were obtained from Ibidi.

### Chitosan preparation.

Chitosan was subjected to several filtrations in order to remove insolubles and impurities before any use. Chitosan was first solubilized in an AcOH aqueous solution, followed by successive filtrations through cellulose membrane (Millipore), with pore sizes ranging from 3 to 0.22 μm. Chitosan was then precipitated with ammonium hydroxide and washed by centrifugation with deionized water until a neutral pH was obtained. The purified chitosan was finally lyophilized and stored at room temperature.

In order to investigate the effect of DA on the film properties, chitosans with various DA were prepared by chemical modification using acetic anhydride for both chitosans of different molar masses (18). Chitosan was first dissolved in an AcOH aqueous solution (1% [wt/wt]) overnight. A mixture of acetic anhydride and 1,2-propanediol was then added dropwise in the chitosan solution for at least 12 h under mechanical stirring. The amount of acetic anhydride added was calculated according to the DA aimed. The final solution was finally washed and lyophilized in the same manner as after the filtration step. The DA of the different chitosans prepared was determined by ^1^H NMR (Bruker Advance III [400 MHz]). For chitosan_156_, the DA obtained are 9.0, 14.5, 25.6, 35.3, 41.9, and 52.2%. DA close to those obtained for chitosan_156_ were obtained for chitosan_557_: 8.0, 12.2, 21.5, 34.0, 45.3, and 52.6%.

### Film preparation.

Silicon substrates and glass coverslips were cleaned from organic pollution using a piranha bath (H_2_SO_4_-H_2_O_2_ at 7/3 [vol/vol]), heated at 150°C for 15 min, and then rinsed with deionized water (resistivity of 18 MΩ.cm). They were then subject to ultrasonication in deionized water for 15 min and dried under a flux of clean air. The substrates (glass or silicon) were then placed into a plasma cleaner (Harrick Plasma) for 15 min in order to generate the silanol groups at the surface for a better adsorption of chitosan polymer chains.

In the meantime, chitosan was solubilized overnight in a solution of deionized water (Otec) with AcOH under magnetic stirring and at room temperature. The amount of acid added was calculated in stoichiometry compared to amine groups available along the chitosan polymer chain. Chitosan solutions with different concentrations ranging from 0.3% to 1% for chitosan_557_ and concentrations ranging from 0.5% to 2% for chitosan_156_ were investigated in this study.

The films were finally formed onto glass substrates by spin-coating at 2,000 rpm until the solvent evaporates completely (5 min). After spin-coating, films were stored 24 h at room temperature before being characterized (unless described otherwise). Some of the films were finally rinsed in AcOH aqueous solution (pH 4) for 5 min so that only the adsorbed chains of chitosan remain on the sample; the samples were then immersed in a water bath and finally dried under a flux of clean air (at a thickness of <3 nm in all cases).

### Surface topography.

The surface morphologies were observed by atomic force microscopy (AFM [CSI Nano-observer]). AFM probes with spring rate close to 40 N/m were purchased from Bruker. The AFM images were processed using Gwyddion software.

### Thickness measurement.

The film thickness was measured on the silicon wafers using spectroscopic ellipsometry. On glass coverslips, the measurements were carried out using profilometry on scratched films. The consistency of the results obtained by ellipsometry or using profilometer profiles independently of the substrate used for a given chitosan solution permitted the use of measurement by ellipsometry as a reference.

The ellipsometer (Sopra GES-5E) was set at an incident angle of 70°, very close to the silicon Brewster angle. At least three measurements were done on each film at different positions in order to verify the film homogeneity. Data were then processed using WINELLI (Sopra-SA) software. A Cauchy model was used to fit experimental data (cos Δ, tan Ψ), in the spectral range of 2.0 to 4.5 eV, depending on fits and regression qualities, to evaluate the thickness. The UV parameters *A* and *B* were respectively, set to 1.53 and 0.002.

A mechanical profilometer (Veeco Instruments) equipped with a cantilever of 2.5 μm in diameter was used to measure film thickness on glass coverslips. For this purpose, the samples were previously scratched with tweezers to locally remove the chitosan film. Data analysis was performed with VISION V4.10 software from Veeco Instruments.

### Wetting measurements.

Contact angles were measured using a tensiometer kit (Easydrop; Krüss GmbH) with a camera connected to a computer equipped with a drop shape analysis software. To put down the liquid drop on the surface, a Hamilton syringe of 1 ml and a needle with a diameter of 0.5 mm were used. “Static” measurements correspond to the angle determined 10 s after water drop deposition.

### Strains, cell cultures, and preparation of media.

The strains used were either lab strains (K-12 and MG1655) or clinical strains obtained from the Laboratoire de Biologie—Centre Hospitalier Martigues.

E. coli and K. pneumoniae cells were grown in ion-adjusted Luria-Bertani (LB) medium until exponential phase (optical density [OD] = 0.5 ± 0.1) and diluted in LB to an OD of around 0.01. The LB medium was prepared using 10 g/liter Bacto-Casitone (BD; 225930), 5 g/liter NaCl (Biosciences; RC-093), 5 g/liter Bacto yeast extract (BD; 212750), and osmosed water supplemented with 0.46 μg/liter MgCl_2_, 2.31 μg/liter CaCl_2_, 5.02 μg/liter ZnCl_2_, and 6.15 μg/liter KCl. The cell suspension was then directly added to microfluidic channels. Loaded microfluidic chambers were centrifuged for 3 min at 1,000 rcf (Eppendorf centrifuge 5430R) to maximize cell adhesion.

### Preparation of chitosan slides and microfluidic chambers.

Microfluidic channels were prepared from commercially available six-channel systems (sticky-slide VI 0.4; Ibidi) that were directly applied to the surface of chitosan-coated slides (CCSs). The dried chitosan was rehydrated by addition of deionized Milli-Q water for at least 5 min.

After centrifugation, the microfluidic channels were connected to a syringe and a pump (Aladdin syringe pump WPI). The remaining nonadherent cells were thus removed through rinse steps: a 1.5-ml rinse with a 1.5-ml/min flow followed by 1.5 ml with a 5-ml/min flow. The work flow was set at 3 ml/h. Adhesion strength was assessed by increasing the flow in the channel. The shear stress was calculated by the formula given by Ibidi τ = η·176.1·Φ, where τ is the shear stress (dyn/cm^2^), η is the dynamical viscosity (dyn.s/cm^2^), and Φ is the flow rate (ml/min). In the absence of data about LB dynamical viscosity, we hypothesize that it is close to that of the cell culture medium, which is around 0.0072 dyn·s/cm^2^. On C5, adhered cells resisted shear forces above 12.3 dyn/cm^2^, indicating that they were firmly adhered.

### Dyes, antibiotic treatment, and MIC determination.

Propidium iodide (PI) is a DNA stain that cannot cross the membrane of live cells, making it useful to differentiate healthy cells from dead cells. E. coli cells were immobilized to C5 chitosan on the microfluidic chamber in the presence or absence of antibiotics. Immediately before acquisition, the channel was rinsed with LB supplemented with 3 mg/liter ertapenem (Sigma-Aldrich) and 50 μl/ml PI (Sigma-Aldrich; P4170).

For MIC determination, different channels were prepared simultaneously with the same cell suspension. The antibiotics ertapenem, ampicillin (Sigma-Aldrich), amdinocillin (Sigma-Aldrich), and oxofloxacin (Sigma-Aldrich) were prepared at different concentrations (one channel contained only LB as a control) and added to each channel just before image acquisition (every 3 min for standard acquisition). The MIC was defined for the lowest antibiotic concentration that induced cell death/stasis.

### Microscope acquisition and image manipulation.

Images were acquired with a Nikon phase-contrast microscope (TE2000) equipped with a motorized stage, a Nikon perfect focus system, and a 100× lens objective. For technical convenience, experiments were performed at 25°C, a condition that supported growth of both E. coli and K. pneumoniae. Standard image analyses were performed under MicrobeJ, a Fiji plug-in developed for the analysis of bacteria (31).

### RICM.

RICM was performed with a Zeiss Observer inverted microscope (Carl Zeiss, Jena, Germany) equipped with a Zeiss Neofluar 63/1.25 antiflex objective, a crossed-polarizers cube, and a C7780 camera (Hamamatsu, Tokyo, Japan) with an adjustable field and aperture stops. The source was an X-cite 120Q lamp (Exfo, Mississauga, Canada) coupled to a narrow-bandpass filter (λ = 546 ± 12 nm).

### Image segmentation.

Image segmentation procedures were developed in Python. In order to provide a streamlined analysis procedure, we used the parameter-free threshold setting algorithm “iso_data” from the scikit-image Python package (32) to extract the contours of the bacterial cells.

For each contour, we then perform a singular value decomposition from the NumPy library (33) to retrieve aligned and centered contours for each bacteria. We use defect analysis (provided by the OpenCV library) to detect the septum and split the contours. If the defects attributed to the septum have a distance of less than 0.5 μm and their center is less than 0.3 μm from the cell center, the contour is considered to be composed of two cells and is therefore split.

From the detected and split contours, we then extract relevant morphometric data:

• The contour area
• The contour length or perimeter
• The length of the minimum-area rectangle
• The width of the minimum-area rectangle
• The circularity defined as 4π*A*/*ℓ*^2^: equal to 1 if the contour is perfectly circular and lower than 1 otherwise
• The inverse of the aspect ratio of the enclosing rectangle (width/length), always lower than 1
• The ratio of the minimal rectangle area to the cell area (which should be close to 1 for a wild-type rod-shaped cell)

### Image annotation and training.

In order to constitute a training set to apply supervised machine learning, we developed a web-based dashboard based on plotly-dash toolset (http://plot.ly/dash ). The annotation tool allows classification of the detected contours into 5 categories: normal, divided, abnormal, dead, and invalid. We annotated 7 assays corresponding to 8,300 contours. Typically our assays corresponded to microscopy fields of 10 to 20 cells treated with ampicillin.

### Outlier detection.

From the annotated contours, those marked as “normal” were used to train a single-class scalable vector machine classifier provided by the scikit-learn library (34). More precisely, we fit a OneClassSVM object over 75% of the annotated data and used the remaining 25% over the above-defined morphometric data. The trained classifier was then used on all the detected data to remove invalid contours from the count on each image. Ampicillin generates a number of morphological aberrations that deviate from untreated cells and thus provided a sensitive test to detect early defects induced by antibiotics that lead to abnormal morphologies. This training was also efficient to determine susceptibility to other antibiotics that cause morphological defects (i.e., ertapenem).

### Sensitivity criterion.

For each assay, the growth rate (*G*) was computed by performing a linear regression of the logarithm of the number of detected bacteria versus time:

$$N(t)=N_{0}\;2^{t/{\text{δ}}_{t}}\Leftrightarrow {\text{log}}_{2}N(t)={\text{log}}_{2}N_{0}+t/{\text{δ}}_{t}$$

The reported error is the 95% confidence interval. We used the scipy.stats.theilslopes method (35) to perform the linear regression. A given growth assay was considered to survive if the growth rate was 0.2 h^−1^. This corresponds to a doubling time (δ) of δ = ln(2)/*G* lower than 200 min. This cutoff was chosen as it is longer than the microscopy acquisition span (Fig. S2D).

## References

[1] Cattoni DI, Fiche J-B, Valeri A, Mignot T, Nöllmann M 2013 Super-resolution imaging of bacteria in a microfluidics device. PLoS One 8:e76268. doi:10.1371/journal.pone.0076268. 24146850 PMC3797773

[2] Baltekin Ö, Boucharin A, Tano E, Andersson DI, Elf J 2017 Antibiotic susceptibility testing in less than 30 min using direct single-cell imaging. Proc Natl Acad Sci U S A 114:9170–9175. doi:10.1073/pnas.1708558114. 28790187 PMC5576829

[3] Choi J, Yoo J, Lee M, Kim E-G, Lee JS, Lee S, Joo S, Song SH, Kim E-C, Lee JC, Kim HC, Jung Y-G, Kwon S 2014 A rapid antimicrobial susceptibility test based on single-cell morphological analysis. Sci Transl Med 6:267ra174. doi:10.1126/scitranslmed.3009650. 25520395

[4] Ducret A, Maisonneuve E, Notareschi P, Grossi A, Mignot T, Dukan S 2009 A microscope automated fluidic system to study bacterial processes in real time. PLoS One 4:e7282. doi:10.1371/journal.pone.0007282. 19789641 PMC2748647

[5] Matsumoto Y, Sakakihara S, Grushnikov A, Kikuchi K, Noji H, Yamaguchi A, Iino R, Yagi Y, Nishino K 2016 A microfluidic channel method for rapid drug-susceptibility testing of Pseudomonas aeruginosa. PLoS One 11:e0148797. doi:10.1371/journal.pone.0148797. 26872134 PMC4752270

[6] Strahl H, Hamoen LW 2010 Membrane potential is important for bacterial cell division. Proc Natl Acad Sci U S A 107:12281–12286. doi:10.1073/pnas.1005485107. 20566861 PMC2901462

[7] Katsu T, Tsuchiya T, Fujita Y 1984 Dissipation of membrane potential of Escherichia coli cells induced by macromolecular polylysine. Biochem Biophys Res Commun 122:401–406. doi:10.1016/0006-291X(84)90489-3. 6378203

[8] Colville K, Tompkins N, Rutenberg AD, Jericho MH 2010 Effects of poly(l-lysine) substrates on attached Escherichia coli bacteria. Langmuir 26:2639–2644. doi:10.1021/la902826n. 19761262

[9] Ezraty B, Vergnes A, Banzhaf M, Duverger Y, Huguenot A, Brochado AR, Su S-Y, Espinosa L, Loiseau L, Py B, Typas A, Barras F 2013 Fe-S cluster biosynthesis controls uptake of aminoglycosides in a ROS-less death pathway. Science 340:1583–1587. doi:10.1126/science.1238328. 23812717

[10] Lutgring JD, Bittencourt C, McElvania TeKippe E, Cavuoti D, Hollaway R, Burd EM 2018 Evaluation of the Accelerate Pheno system: results from two academic medical centers. J Clin Microbiol 56:e01672-17. doi:10.1128/JCM.01672-17. 29386262 PMC5869849

[11] Pancholi P, Carroll KC, Buchan BW, Chan RC, Dhiman N, Ford B, Granato PA, Harrington AT, Hernandez DR, Humphries RM, Jindra MR, Ledeboer NA, Miller SA, Mochon AB, Morgan MA, Patel R, Schreckenberger PC, Stamper PD, Simner PJ, Tucci NE, Zimmerman C, Wolk DM 2018 Multicenter evaluation of the Accelerate PhenoTest BC kit for rapid identification and phenotypic antimicrobial susceptibility testing using morphokinetic cellular analysis. J Clin Microbiol 56:e01329-17. doi:10.1128/JCM.01329-17. PMC5869823 29305546

[12] Metzger SW, Howson DC, Goldberg DA, Buttry DA March 2008 Rapid microbial detection and antimicrobial susceptibility testing. US patent 7,341,841 B2.

[13] Raafat D, Sahl H 2009 Chitosan and its antimicrobial potential—a critical literature survey. Microb Biotechnol 2:186–201. doi:10.1111/j.1751-7915.2008.00080.x. 21261913 PMC3815839

[14] Santajit S, Indrawattana N 2016 Mechanisms of antimicrobial resistance in ESKAPE pathogens. Biomed Res Int 2016:2475067. doi:10.1155/2016/2475067. 27274985 PMC4871955

[15] Foster LJR, Ho S, Hook J, Basuki M, Marçal H 2015 Chitosan as a biomaterial: influence of degree of deacetylation on its physiochemical, material and biological properties. PLoS One 10:e0135153. doi:10.1371/journal.pone.0135153. 26305690 PMC4549144

[16] Nunthanid J, Puttipipatkhachorn S, Yamamoto K, Peck GE 2001 Physical properties and molecular behavior of chitosan films. Drug Dev Ind Pharm 27:143–157. doi:10.1081/DDC-100000481. 11266226

[17] Domard A 2011 A perspective on 30 years research on chitin and chitosan. Carbohydr Polym 84:696–703. doi:10.1016/j.carbpol.2010.04.083.

[18] Vachoud L, Zydowicz N, Domard A 1997 Formation and characterisation of a physical chitin gel. Carbohydr Res 302:169–177. doi:10.1016/S0008-6215(97)00126-2.

[19] Drescher K, Dunkel J, Nadell CD, van Teeffelen S, Grnja I, Wingreen NS, Stone HA, Bassler BL 2016 Architectural transitions in Vibrio cholerae biofilms at single-cell resolution. Proc Natl Acad Sci U S A 113:E2066–E2072. doi:10.1073/pnas.1601702113. 26933214 PMC4833255

[20] Faure LM, Fiche J-B, Espinosa L, Ducret A, Anantharaman V, Luciano J, Lhospice S, Islam ST, Tréguier J, Sotes M, Kuru E, Van Nieuwenhze MS, Brun YV, Théodoly O, Aravind L, Nollmann M, Mignot T 2016 The mechanism of force transmission at bacterial focal adhesion complexes. Nature 539:530–535. doi:10.1038/nature20121. 27749817 PMC5465867

[21] Michelson AD 2002 Platelets. Gulf Professional Publishing, Houston, TX.

[22] Yao Z, Kahne D, Kishony R 2012 Distinct single-cell morphological dynamics under beta-lactam antibiotics. Mol Cell 48:705–712. doi:10.1016/j.molcel.2012.09.016. 23103254 PMC3525771

[23] Stapels DAC, Hill PWS, Westermann AJ, Fisher RA, Thurston TL, Saliba A-E, Blommestein I, Vogel J, Helaine S 2018 Salmonella persisters undermine host immune defenses during antibiotic treatment. Science 362:1156–1160. doi:10.1126/science.aat7148. 30523110

[24] Rasko DA, Rosovitz MJ, Myers GSA, Mongodin EF, Fricke WF, Gajer P, Crabtree J, Sebaihia M, Thomson NR, Chaudhuri R, Henderson IR, Sperandio V, Ravel J 2008 The pangenome structure of Escherichia coli: comparative genomic analysis of E. coli commensal and pathogenic isolates. J Bacteriol 190:6881–6893. doi:10.1128/JB.00619-08. 18676672 PMC2566221

[25] Duvernoy M-C, Mora T, Ardré M, Croquette V, Bensimon D, Quilliet C, Ghigo J-M, Balland M, Beloin C, Lecuyer S, Desprat N 2018 Asymmetric adhesion of rod-shaped bacteria controls microcolony morphogenesis. Nat Commun 9:1120. doi:10.1038/s41467-018-03446-y. 29549338 PMC5856753

[26] Sciarretta K, Røttingen J-A, Opalska A, Van Hengel AJ, Larsen J 2016 Economic incentives for antibacterial drug development: literature review and considerations from the Transatlantic Task Force on Antimicrobial Resistance. Clin Infect Dis 63:1470–1474. doi:10.1093/cid/ciw593. 27578820

[27] Ling TKW, Tam PC, Liu ZK, Cheng AFB 2001 Evaluation of VITEK 2 rapid identification and susceptibility testing system against Gram-negative clinical isolates. J Clin Microbiol 39:2964–2966. doi:10.1128/JCM.39.8.2964-2966.2001. 11474023 PMC88270

[28] Reller LB, Weinstein M, Jorgensen JH, Ferraro MJ 2009 Antimicrobial susceptibility testing: a review of general principles and contemporary practices. Clin Infect Dis 49:1749–1755. doi:10.1086/647952. 19857164

[29] Dai J, Hamon M, Jambovane S 2016 Microfluidics for antibiotic susceptibility and toxicity testing. Bioengineering 3:25. doi:10.3390/bioengineering3040025. PMC5597268 28952587

[30] Mignot T, Theodoly O, David L, Sudre G 30 5 2018, filing date A film of chitosan and a device comprising the same deposited on a substrate and uses thereof. Priority European patent application EP18305666.2.

[31] Ducret A, Quardokus EM, Brun YV 2016 MicrobeJ, a tool for high throughput bacterial cell detection and quantitative analysis. Nat Microbiol 1:16077. doi:10.1038/nmicrobiol.2016.77. 27572972 PMC5010025

[32] van der Walt S, Schönberger JL, Nunez-Iglesias J, Boulogne F, Warner JD, Yager N, Gouillart E, Yu T, scikit-image contributors. 2014 scikit-image: image processing in Python. PeerJ 2:e453. doi:10.7717/peerj.453. 25024921 PMC4081273

[33] van der Walt S, Colbert SC, Varoquaux G 2011 The NumPy array: a structure for efficient numerical computation. Comput Sci Eng 13:22–30. doi:10.1109/MCSE.2011.37.

[34] Pedregosa F, Varoquaux G, Gramfort A, Michel V, Thirion B, Grisel O, Blondel M, Prettenhofer P, Weiss R, Dubourg V, Vanderplas J, Passos A, Cournapeau D, Brucher M, Perrot M, Duchesnay É 2011 Scikit-learn: machine learning in Python. J Mach Learn Res 12:2825–2830.

[35] Sen PK 1968 Estimates of the regression coefficient based on Kendall’s tau. J Am Stat Assoc 63:1379–1389. doi:10.2307/2285891.

