# Supplementary figures and images for: Chitosan Films for Microfluidic Studies of Single Bacteria and Perspectives for Antibiotic Susceptibility Testing
Source: mBio. 2019 Aug 20;10(4):e01375-19. doi: 10.1128/mBio.01375-19 (PMC6703423; doi:10.1128/mBio.01375-19)

**A**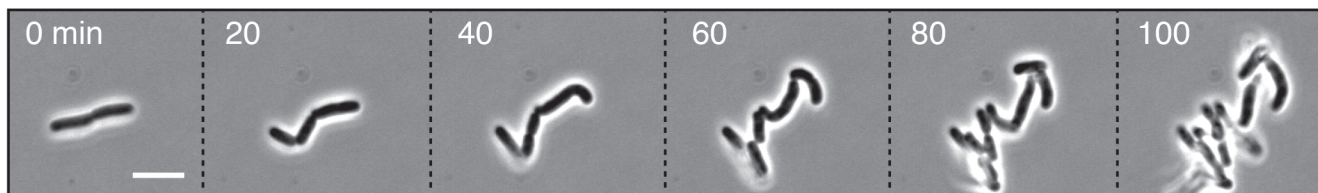**B**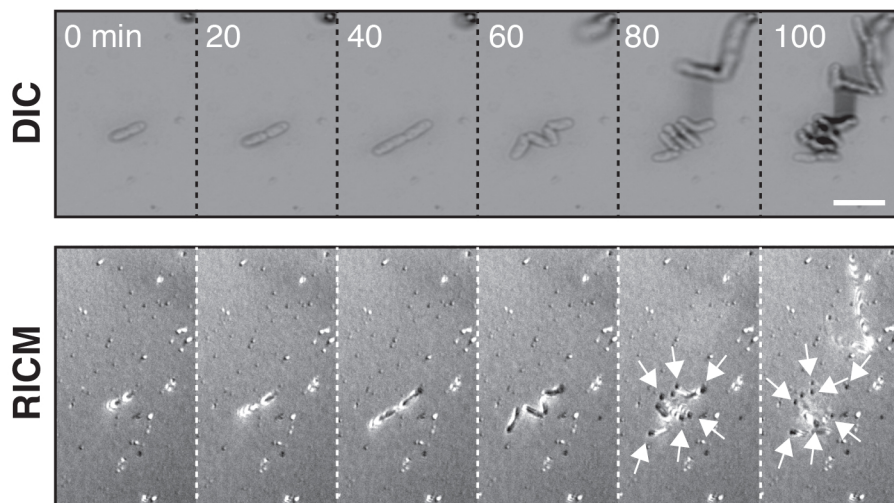**C**

Leave culture 3 days and add fresh culture medium

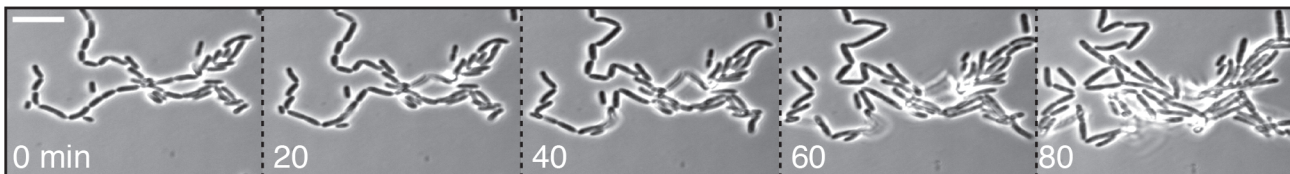

Supplement: FIG S1 [file mBio.01375-19-sf001.pdf]

**A**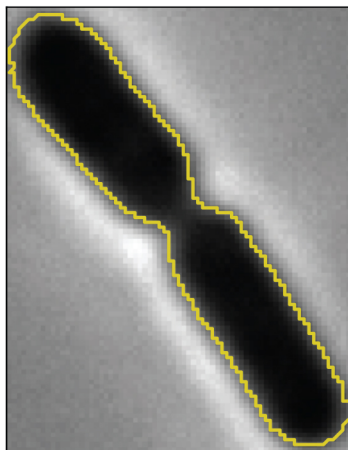**B**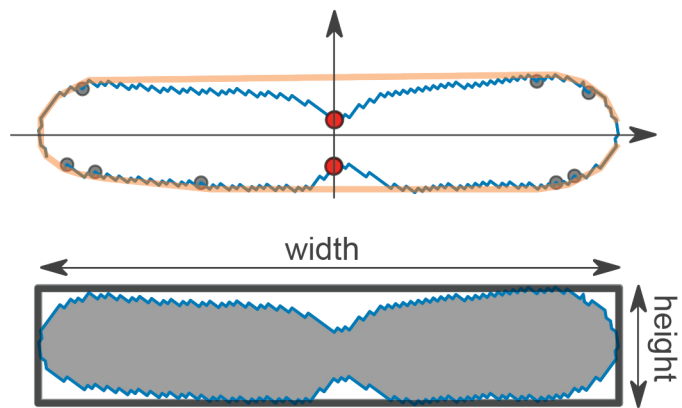**C**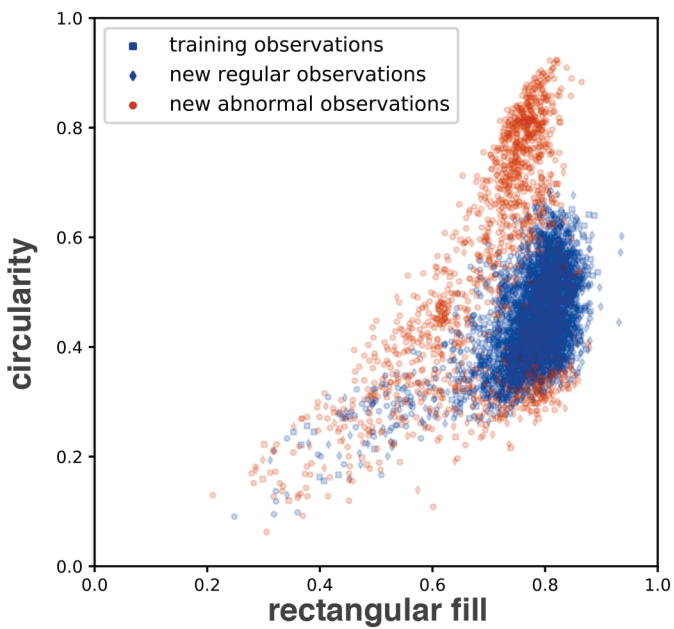

Supplement: FIG S2 [file mBio.01375-19-sf002.pdf]

0 min

30 min

60 min

90 min

120 min

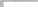

Supplement: FIG S3 [file mBio.01375-19-sf003.pdf]

0 min

75 min

150 min

225 min

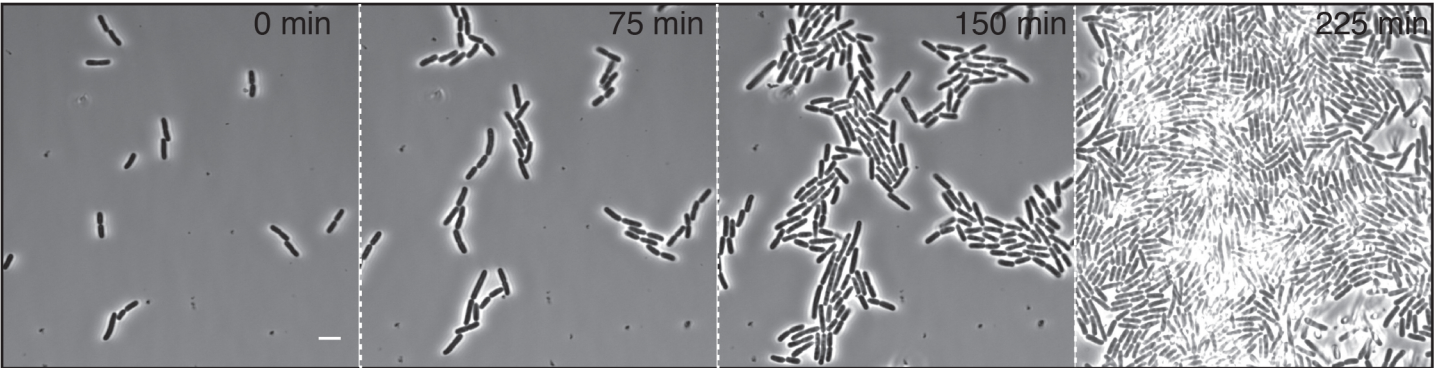

Supplement: FIG S4 [file mBio.01375-19-sf004.pdf]
